# The Effect of Phosphoric Acid on the Preparation of High-Performance Li_3_InCl_6_ Solid-State Electrolytes by Water-Mediated Synthesis

**DOI:** 10.3390/ma18092077

**Published:** 2025-05-01

**Authors:** Shuqing Wen, Hualin Sheng, Songsheng Zheng, Zhaolin Wang

**Affiliations:** College of Energy, Xiamen University, Xiamen 361002, China; shuqing@stu.xmu.edu.cn (S.W.); shenghl@stu.xmu.edu.cn (H.S.)

**Keywords:** solid-state electrolytes, ionic conductivity, water-mediated synthesis, phosphoric acid

## Abstract

The halide solid-state electrolyte Li_3_InCl_6_ (LIC) is an excellent solid-state electrolyte for lithium-ion batteries. In this study, phosphoric acid (H_3_PO_4_) solution was added during the preparation of Li_3_InCl_6_ to obtain LIC samples with high ionic conductivity, and the effect of different concentrations of H_3_PO_4_ on increasing the ionic conductivity of LIC was investigated. The results showed that the LIC prepared in 1% H_3_PO_4_ had the least impurities and the highest ionic conductivity of 1.15 × 10^−3^ S/cm, and the all-solid-state LiCoO_2_-LIC/LIC-1%H_3_PO_4_/Li_6_PS_5_Cl/In/Li batteries assembled with this sample could be stably cycled for 14 cycles at 30 °C, presenting a high initial charge capacity of 128 mAh/g at 0.05 C. The Li-In/LPSC/LIC-1% H_3_PO_4_/LPSC/Li-In symmetric cell can be stably cycled at current densities of 0.05 mA/cm^2^, 0.1 mA/cm^2^, and 0.3 mA/cm^2^. Further studies showed that LIC samples prepared in H_3_PO_4_ contained small amounts of LiIn(P_2_O_7_). The structure of LiIn(P_2_O_7_) is a continuous skeleton containing a large number of vacancies for Li^+^ transport, thus improving the ionic conductivity of LIC.

## 1. Introduction

Due to the flammability of traditional liquid lithium-ion batteries, liquid leakage, and other safety issues, the use of solid-state electrolytes (SSEs) instead of liquid electrolytes and separators in the all-solid-state lithium-ion batteries (ASSLIBs) has become the most promising development in next-generation energy storage devices [1,2]. Solid-state electrolytes combine the lithium-ion transport capability of traditional liquid electrolytes with the separator function to isolate electrodes, effectively suppressing lithium dendrite penetration and short-circuit-induced fire hazards [3].

Solid-state electrolytes are categorized into organic polymer solid-state electrolytes and inorganic solid-state electrolytes. The latter further includes oxide solid-state electrolytes, sulfide solid-state electrolytes, and halide solid-state electrolytes [3]. Polymer solid-state electrolytes exhibit favorable mechanical processing properties, but their ionic conductivity is generally low (room-temperature ionic conductivity: 10^−8^–10^−5^ S/cm) [4]. Oxide solid-state electrolytes demonstrate excellent air stability and electrochemical stability; however, they suffer from poor mechanical processability and require high-temperature sintering above 1000 °C for preparation [5]. Sulfide solid-state electrolytes possess high ionic conductivity and relatively good mechanical processing performance, but they generate toxic H_2_S gas when exposed to air [6]. Halide solid-state electrolytes, in contrast, not only achieve high ionic conductivity at room temperature but also avoid releasing toxic gases into the air, positioning them as promising competitors for next-generation solid-state electrolytes [7].

In 2019, Li et al. reported a halide solid-state electrolyte, Li_3_InCl_6_ (LIC), which exhibits an ionic conductivity of 1.49 × 10^−3^ S/cm and can be synthesized at scale in water, paving the way for the industrialization of halide solid-state electrolytes [8]. However, during the dehydration process in the water-mediated synthesis of Li_3_InCl_6_, the material is prone to oxidation, leading to a rapid decline in ionic conductivity [9]. Acidic environments can suppress hydrolysis and the oxidation of the sample [10], and mixed crystals of Li_3_PO_4_ and Li_4_P_2_O_7_ have also been identified as promising solid-state electrolytes [11]. Macarie et al. have demonstrated that phosphonic groups exhibit favorable properties in water-mediated synthesis methods by effectively suppressing the formation of scale-forming salts [12] and investigated the synthesis methods of phosphate compounds [13]. Iliescu et al. demonstrates that phosphorus can enhance the ionic conductivity of polymer solid-state electrolytes [14]. Phosphoric acid was selected as an additive due to its ability to suppress hydrolysis via pH modulation and generation of phosphate derivatives (e.g., P_2_O_7_^4−^) that enhance ionic conductivity.

This study employs water-mediated synthesis to prepare LIC, during which phosphoric acid (H_3_PO_4_) solutions of varying concentrations are introduced to suppress hydrolysis and oxidation of the sample while simultaneously generating a small amount of LiIn(P_2_O_7_) to form a composite with LIC, thereby enhancing the ionic conductivity of the material. Furthermore, a full solid-state battery LiCoO_2_-LIC/LIC-1%H_3_PO_4_/Li_6_PS_5_Cl/In/Li was assembled to evaluate its cycling performance.

## 2. Materials and Methods

### 2.1. Materials Synthesis

**Figure 1:**
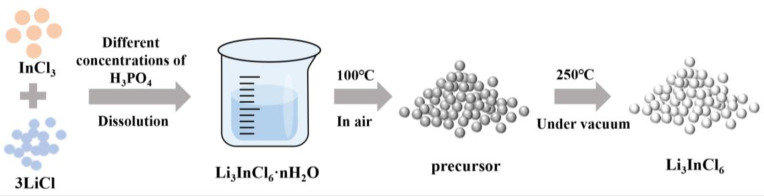
Synthesis methodology of Li_3_InCl_6_ powder.

As depicted in Figure 1, precursor powders of LiCl (99%, Tianjin Xiensi, Tianjin, China) and InCl_3_ (98%, Macklin, Shanghai, China) were precisely weighed at a 3:1 molar ratio and mechanically homogenized via agate mortar grinding. The resulting mixture was dissolved in phosphoric acid (H_3_PO_4_, Hushi, Shanghai, China, 85.0%) solutions with mass concentrations of 0%, 0.5%, 1%, and 2%, respectively. H_3_PO_4_ concentrations (0–2%) were chosen to systematically investigate the trade-off between crystalline water suppression and LiIn(P_2_O_7_) over formation. According to the water-mediated synthesis route for Li_3_InCl_6_ reported by Li et al. [8], the solutions were dried in a forced-air drying oven at 100 °C for 4 h to obtain hydrated precursors. To prevent oxidation, the hydrated precursors were ground into powder under an inert atmosphere in a glovebox and subsequently dried in a vacuum-drying oven at 250 °C for 4 h, yielding the final Li_3_InCl_6_ powders. Samples synthesized in pure water and H_3_PO_4_ solutions of varying concentrations were labeled as LIC-H_2_O, LIC-0.5% H_3_PO_4_, LIC-1% H_3_PO_4_, and LIC-2% H_3_PO_4_.

### 2.2. Structural Characterization

X-ray diffraction analysis (Rigaku Ultima IV, 2θ range: 10–80°, scan rate: 10°/min) was employed to characterize the crystallographic phase composition of the specimens. The micro-morphology of the samples was characterized using a scanning electron microscope (SEM; Hitachi TM3030, Tokyo, Japan). The samples were mixed with KBr at a mass ratio of 1:100, and the infrared spectra of the samples were obtained using a Nicolet Is5 FTIR spectrometer (Thermo Fisher Scientific, Waltham, MA, USA). Simultaneous TG-DSC measurements (Netzsch STA449F5, Selb, Germany) were conducted under dynamic heating (25–400 °C, 15 °C/min) in a nitrogen atmosphere (flow rate: 80 mL/min) to probe thermal stability. The TG temperature range was capped at 400 °C to prevent thermal decomposition of LiCl and InCl_3_ [15].

### 2.3. Electrochemical Measurements

**Figure 2:**
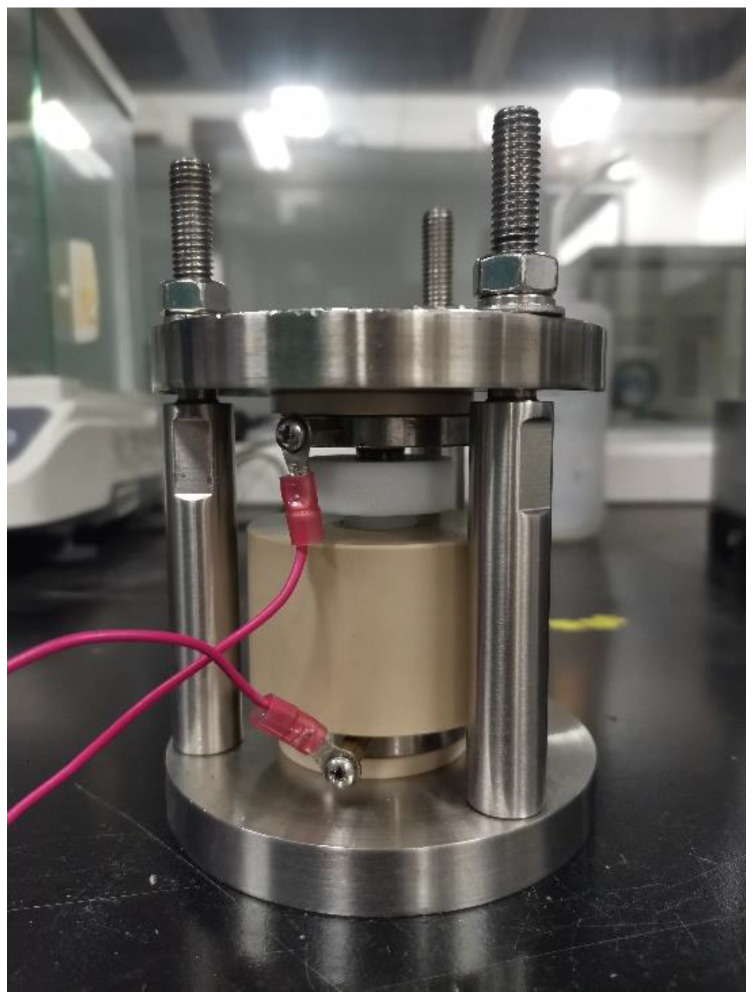
PEEK (polyetheretherketone) molds for solid electrolytes.

Electrochemical impedance spectroscopy (EIS) measurements were conducted on the synthesized LIC powder using a Metrohm Autolab PGSTAT204 workstation (Ota, Japan). Moreover, 0.15 g of LIC powder was placed between two stainless steel rods of 10 mm diameter in a PEEK (polyether ether ketone) mold (Figure 2), and a pressure of 300 MPa was applied to form a symmetric cell of the powder in the mold. EIS measurements were conducted under an argon atmosphere at ambient temperature (25 °C) across a frequency range of 0.01 Hz to 1 MHz to assess interfacial and bulk resistance. Subsequently, cyclic voltammetry (CV) was performed using a electrochemical workstation (Shanghai CHI760E, CH Instruments, Shanghai, China) on asymmetric cells with a configuration of Li-In/Li_6_PS_5_Cl/LIC/stainless steel, which were encapsulated in polyetheretherketone (PEEK) molds. The CV scans spanned a potential window of −1 to 3 V (vs. Li^+^/Li-In) at a controlled sweep rate of 0.025 V/s, enabling characterization of redox behavior and interfacial stability. The Blue Battery Test System (LAND-CT2001A, Wuhan Baxter Bao, Wuhan, China) was programmed to cycle cells between 1.9 V and 3.8 V (Li^+^/Li-In) with thermal regulation maintained at 30 ± 0.5 °C.

### 2.4. Assembly of ASSLIBs

The prepared LIC and lithium cobaltate powder (LiCoO_2_, Canrd) were ground uniformly at the mass ratio of 3:7 to obtain the composite cathode powder. The all-solid-state batteries (ASSLIBs) consisted of composite cathode powder, LIC electrolyte powder, Li_6_PS_5_Cl (LPSC, Shenzhen Cogent, Shenzhen, China), In foil (99.999%, Φ = 10 mm, thickness = 0.1 mm) and Li foil (99.95%, Φ = 10 mm, thickness = 0.1 mm). Firstly, 0.15 g of LIC powder was weighed with an electronic balance and added to a PEEK mold, and the powder was pressed into discs in the mold with a pressure of 300 MPa. Then, LPSC powder and composite positive electrode powder were added at both ends of the discs, and 300 MPa pressure was again applied to the sample in the mold. Finally, indium (In) and lithium (Li) foils were positioned at the LPSC interface to construct the multilayer cell architecture (LiCoO_2_-LIC/LIC/LPSC/In/Li).

## 3. Results and Discussion

**Figure 3:**
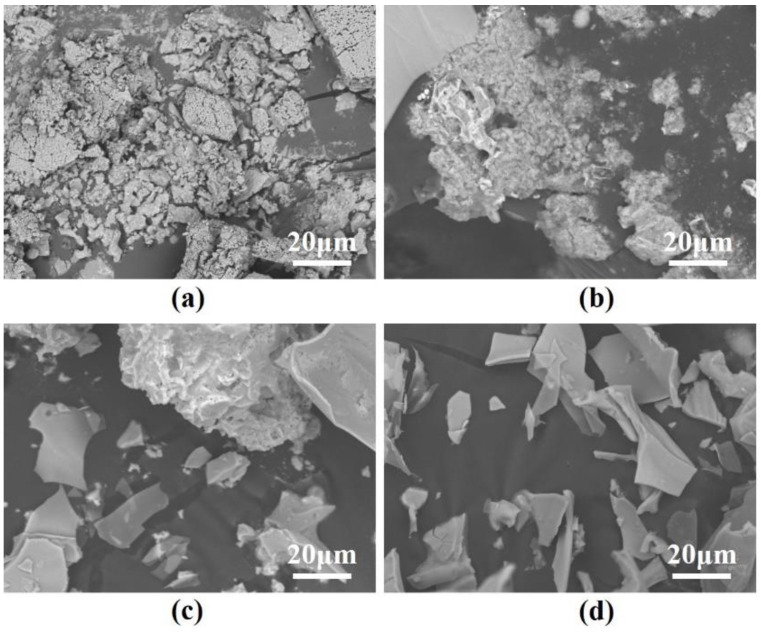
SEM images of LIC prepared in different concentrations of H_3_PO_4_: (**a**) LIC-H_2_O, (**b**) LIC-0.05% H_3_PO_4_, (**c**) LIC-1% H_3_PO_4_, (**d**) LIC-2% H_3_PO_4._

Figure 3 presents the SEM images of dried LIC samples prepared with varying H_3_PO_4_ concentrations. As observed, LIC synthesized in pure water exhibits bulk-like stacking due to the difficulty in removing crystalline water during drying. The LIC-H_2_O sample exhibits bulk-like aggregates with irregular shapes, ranging in size from 10 to 50 μm. When 0.5% H_3_PO_4_ is introduced, the samples form porous bulk structures, which facilitate crystalline water elimination. The porous structure in LIC-0.5% H_3_PO_4_ contains interconnected voids with diameters of 2–10 μm. With increasing H_3_PO_4_ concentration, the microstructure progressively transitions to a flake-like morphology. At 1% H_3_PO_4_, the samples partially display flake-like structures alongside residual porous bulk regions. Finally, at 2% H_3_PO_4_, the microstructure becomes entirely flake-shaped, indicating complete morphological transformation. The microstructure transitions to flake-like particles with lateral dimensions of 5–20 μm and thicknesses of 0.5–2 μm.

**Figure 4:**
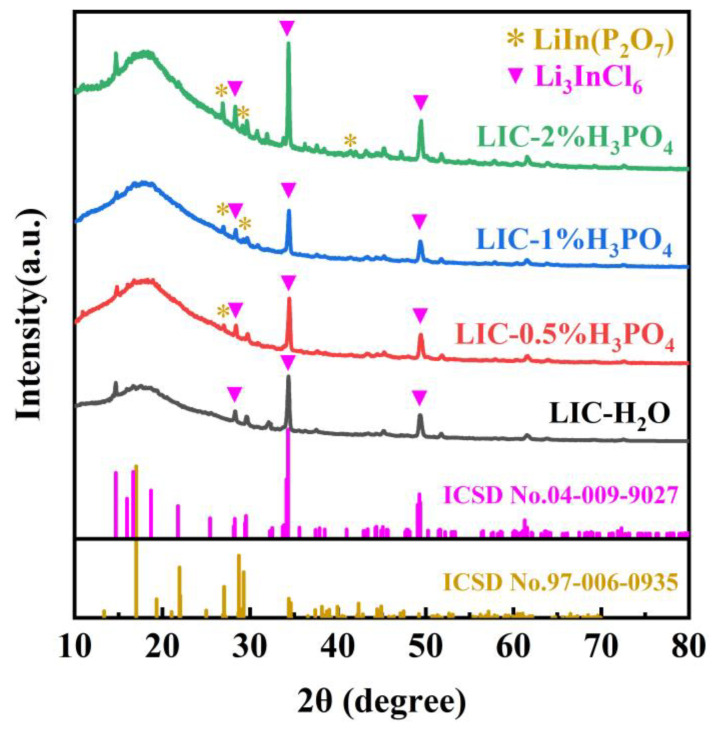
XRD images of LIC prepared in different concentrations of H_3_PO_4._

Figure 4 displays the XRD patterns of LIC powders synthesized with varying H_3_PO_4_ concentrations. During testing, the powders were sealed onto glass slides using Kapton polyimide tape to prevent air exposure. As shown, the XRD patterns of all four samples align well with most characteristic peaks of the LIC reference PDF card (ICSD No. 04-009-9027). The LIC-H_2_O sample (prepared in pure water) exhibits weaker peak intensities compared to others, suggesting lower crystallinity or reduced LIC content. Upon H_3_PO_4_ addition, the XRD patterns reveal coexisting characteristic peaks of LIC and LiIn(P_2_O_7_), confirming the formation of a LIC/LiIn(P_2_O_7_) composite. Notably, the intensity of LiIn(P_2_O_7_) peaks increases progressively with higher H_3_PO_4_ concentrations, indicating enhanced formation of this phase. This is attributed to the thermal decomposition of PO_4_^3−^ (from H_3_PO_4_) into P_2_O_7_^4−^ at 200 °C, which reacts with In^3+^ and Li^+^ in the sample to yield LiIn(P_2_O_7_). The P_2_O_7_^4−^ anion, owing to its favorable structural properties, has been reported to enhance ionic conductivity when composited with solid-state electrolytes [11].

**Figure 5:**
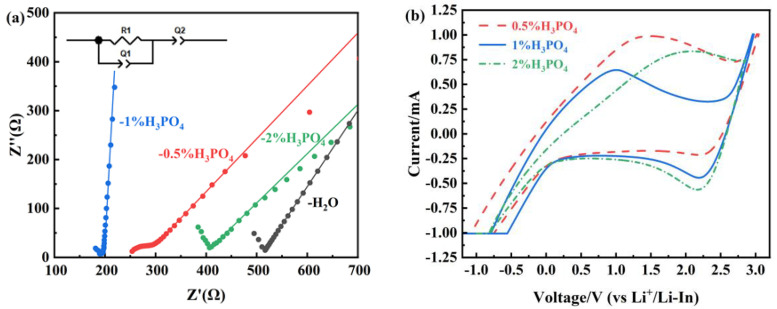
(**a**) Electrochemical impedance plots of LIC prepared in different concentrations of H_3_PO_4_ and (**b**) CV curves of LIC prepared in different concentrations of H_3_PO_4._

The electrochemical impedance spectroscopy (EIS) of the samples, measured under 300 MPa pressure in an Ar atmosphere, is shown in Figure 5a. The inset in the upper-left corner depicts the equivalent circuit based on a simple R(RQ)Q model. By fitting the equivalent circuit and measuring the diameter and thickness of the pressed LIC electrolyte pellets, the ionic conductivity (σ) of the powders was calculated using the following formula:

σ = d/(S × R)(1)

where σ is the ionic conductivity, d is the pellet thickness, R is the measured AC impedance value, and S is the pellet area. The calculated ionic conductivities are summarized in Table 1.

As shown in Table 1, the ionic conductivity follows the order of LIC-1% H_3_PO_4_ > LIC-0.5% H_3_PO_4_ > LIC-2% H_3_PO_4_ > LIC-H_2_O. The LIC-H_2_O sample demonstrates the lowest ionic transport efficiency among the tested materials, with a recorded conductivity of 0.38 × 10^−3^ S/cm. Upon H_3_PO_4_ addition, the ionic conductivity significantly increases, reaching a maximum value of 1.15 × 10^−3^ S/cm for the LIC-1% H_3_PO_4_ sample. The non-linear relationship between H_3_PO_4_ concentration and ionic conductivity (LIC-2% H_3_PO_4_ < LIC-1% H_3_PO_4_) is tied to excessive LiIn(P_2_O_7_) formation, which disrupts the LIC matrix. The ionic conductivity of the sample decreases when the H_3_PO_4_ concentration exceeds 2%, as the inherently low conductivity of LiIn(P_2_O_7_) (~10^−5^ S/cm) cannot compensate for the loss of the highly conductive LIC matrix. Thus, LiIn(P_2_O_7_) functions as a secondary phase rather than a primary conductor, a conclusion consistent with prior studies [11].

Figure 5b displays the cyclic voltammetry (CV) profiles acquired at 0.025 V/s over a potential window of −1.0–3.0 V (vs. Li^+^/Li-In). The LIC-1% H_3_PO_4_ composite demonstrates enhanced peak symmetry relative to counterparts, signifying superior electrochemical reversibility. During anodic polarization, a distinct oxidation peak is observed at 1.0 V, followed by a reduction peak at 2.2 V under cathodic conditions, collectively spanning a broad electrochemical stability window. These results confirm that the electrolyte synthesized with 1% H_3_PO_4_ delivers the best overall performance.

**Figure 6:**
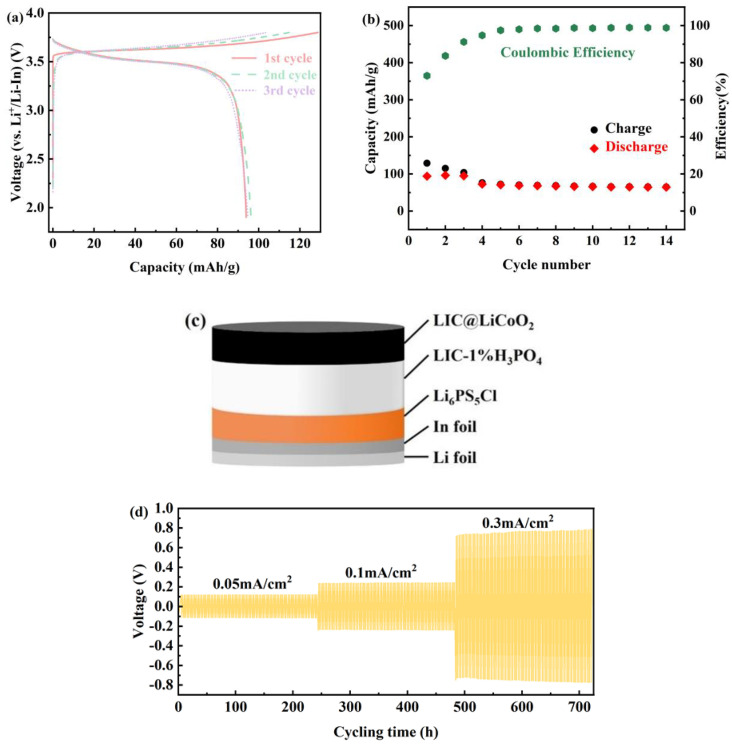
(**a**) Charge–discharge curve of ASSLIBs at 0.05 C magnification, (**b**) cycling performance of ASSLIBs at 0.1 C after initial activation (three cycles at 0.05 C), (**c**) schematic architecture of the ASSLIBs configuration, and (**d**) cyclic stability of symmetric Li-In/LPSC/LIC-1% H_3_PO_4_/LPSC/Li-In cells under varied current densities.

In summary, the LIC-1% H_3_PO_4_ electrolyte demonstrates superior electrochemical performance. To evaluate its practical applicability, the all-solid-state lithium-ion batteries (ASSLIBs) LiCoO_2_-LIC/LIC-1%H_3_PO_4_/LPSC/In/Li were assembled as illustrated in Figure 6c. The cathode comprised a composite of LIC-1% H_3_PO_4_ and LiCoO_2_ blended at a 3:7 weight ratio, while the anode consisted of stacked In and Li foils. LIC-1% H_3_PO_4_ served as the solid electrolyte, with a Li_6_PS_5_Cl interfacial layer inserted between the electrolyte and In foil to mitigate parasitic reactions at the halide–metal interface. The cell fabrication protocol adhered to the methodology outlined in the experimental section. The ASSLIBs underwent three initial activation cycles at 0.05 C within a voltage range of 1.9–3.8 V, followed by charge/discharge cycling at 0.1 C under the same voltage window.

Figure 6a displays the charge–discharge profiles of the ASSLBs at 30 °C under a 0.05 C rate. The first-cycle charge capacity reaches 128 mAh/g with an initial Coulombic efficiency of 72.95%. As shown in Figure 6b, the Coulombic efficiency stabilizes at 98.8% after 14 cycles, after which battery failure occurs due to electrolyte deliquescence in ambient air. Figure 6d presents the cycling performance of a Li-In/LPSC/LIC-1% H_3_PO_4_/LPSC/Li-In symmetric cell under varying current densities. The LPSC interlayers in the symmetric cell configuration are essential for isolating the halide electrolyte from Li metal, ensuring long-term cycling stability [16]. The symmetric cell demonstrates stable cycling over 1000 cycles at 0.05, 0.1, and 0.3 mA/cm^2^, confirming that LIC-1% H_3_PO_4_ maintains stable contact with Li-In metal without detectable side reactions. The LIC-H_2_O sample was excluded from symmetric cell testing due to its inherent instability and low ionic conductivity, which preclude meaningful cycling performance. The superior ionic conductivity of LIC-1% H_3_PO_4_ (1.15 × 10^−3^ S/cm, Table 1) and stable cycling of ASSLIBs (Figure 6a,b) are direct outcomes of phosphorus-enabled composite formation.

**Figure 7:**
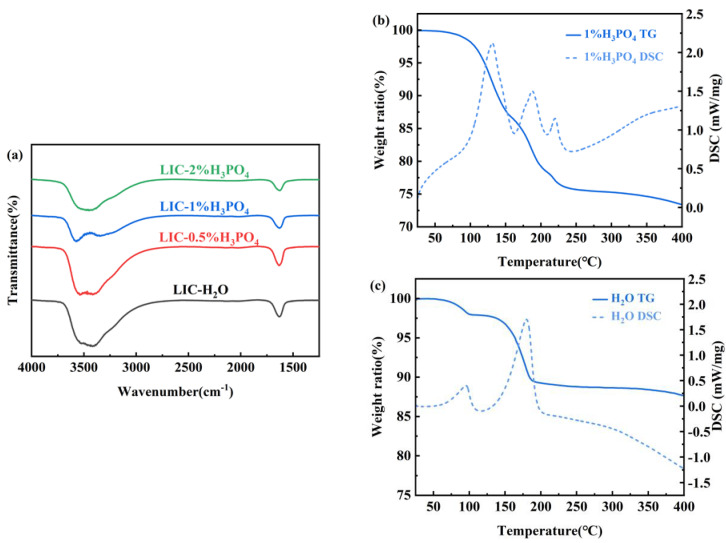
(**a**) Fourier transform infrared images of LIC prepared in different concentrations of H_3_PO_4_, (**b**) thermogravimetric curves of LIC prepared in 1% H_3_PO_4_, and (**c**) thermogravimetric curves of LIC prepared in H_2_O.

Figure 7a shows the Fourier transform infrared (FTIR) spectra of LIC powders synthesized with varying H_3_PO_4_ concentrations. The peak at 3500 cm^−1^ corresponds to hydroxyl group vibrations. The LIC-H_2_O sample exhibits the highest peak intensity at 3500 cm^−1^, indicating the highest crystalline water content in the pure water-synthesized sample. Upon H_3_PO_4_ addition, the hydroxyl peak intensity gradually decreases, reaching its minimum at 1% H_3_PO_4_, which corresponds to the lowest crystalline water content. The progressive reduction of hydroxyl peaks (3500 cm^−1^) with increasing H_3_PO_4_ concentration directly correlates with phosphorus-mediated suppression of crystalline water. However, further increasing the H_3_PO_4_ concentration to 2% results in no significant reduction in hydroxyl peak intensity, suggesting that H_3_PO_4_ concentrations above 1% have limited efficacy in reducing crystalline water. According to Sacci et al. [9], crystalline water in the sample undergoes hydrolysis during thermal dehydration, generating oxide impurities such as InOCl and In_2_O_3_ via the following reactions:

In^3+^ + H_2_O + 3Cl^−^ → InOCl + 2HCl (2)

2In^3+^ + 3H_2_O + 6Cl^−^ → In_2_O_3_ + 6HCl_(g)_ (3)

Therefore, the reduction of the water of crystallization content in the precursor is conducive to obtaining purer LIC samples.

Figure 7b presents the thermogravimetric (TG) curve of the LIC precursor powder synthesized in 1% H_3_PO_4_. The dehydration process concludes at 250 °C, justifying the selection of this temperature for precursor dehydration. The TG curve exhibits three endothermic peaks across the temperature ranges of 100–150 °C, 150–200 °C, and 200–250 °C. The weight loss in the interval of 100–150 °C mainly originates from the free water and H_3_PO_4_ solution not removed from the sample surface, while 150–200 °C is the process of dehydration of the water of crystallization in the precursor samples, in which the precursor Li_3_InCl_6_·nH_2_O removes the water of crystallization to form Li_3_InCl_6_. Since the sample is susceptible to oxidation by the water of crystallization to form In_2_O_3_ impurities during the dehydration process, resulting in a drastic decrease in the ionic conductivity of the sample, a high degree of vacuum must be ensured during the dehydration process. The heat absorption peaks in the range of 200–250 °C correspond to the reaction of PO_4_^3−^ in the sample with the In^3+^ and Li^+^ in the sample to form LiIn(P_2_O_7_). The lack of a 200–250 °C DSC peak in LIC-H_2_O (Figure 7c) confirms that the observed thermal event in H_3_PO_4_-containing samples arises from phosphate-specific reactivity. The dehydration mechanism involving PO_4_^3−^ → P_2_O_7_⁴^−^ transformation is attributed to H_3_PO_4_-derived phosphorus species [17,18]. This process generates LiIn(P_2_O_7_), a key contributor to ionic conductivity enhancement [19].

**Figure 8:**
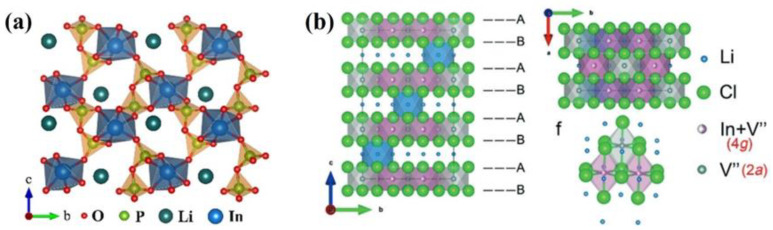
(**a**) Crystal structure of LiIn(P_2_O_7_) [19] and (**b**) crystal structure of LIC [8].

Kartini et al. demonstrated that compositing P_2_O_7_^4−^ with solid-state electrolytes can significantly enhance ionic conductivity due to the exceptional crystal structure of P_2_O_7_^4−^ [11]. LiIn(P_2_O_7_) crystallizes in the triclinic system with the space group P-1, and its crystal structure is illustrated in Figure 8a [19]. In contrast, Li_3_InCl_6_ adopts a monoclinic structure with the space group C2/m, as shown in Figure 8b [8]. The composite of LiIn(P_2_O_7_) and LIC introduces a mixed-anion effect, where In^3+^ ions coordinate with P_2_O_7_^4−^ groups to form a continuous framework rich in vacancies. This framework facilitates efficient Li^+^ ion transport, thereby improving the overall ionic conductivity of the electrolyte. While PO_4_^3−^ reacts with a fraction of In^3+^/Li^+^ to form LiIn(P_2_O_7_), the composite’s synergistic Li^+^ transport mechanisms ensure net conductivity enhancement, as evidenced by the 1.15 × 10^−3^ S/cm value for LIC-1% H_3_PO_4_ (Table 1). In summary, H_3_PO_4_ addition achieves dual objectives; on the one hand, H_3_PO_4_ can minimize hydrolysis-derived impurities (InOCl/In_2_O_3_), and on the other hand, addition of H_3_PO_4_ generates LiIn(P_2_O_7_) with P_2_O_7_^4−^ enabled Li^+^ pathways. The optimal 1% H_3_PO_4_ concentration balances these effects, yielding the highest conductivity.

## 4. Conclusions

This study demonstrates that the addition of H_3_PO_4_ during the water-mediated synthesis of LIC significantly enhances its ionic conductivity, with a systematic investigation into the relationship between H_3_PO_4_ concentration and LIC performance. Experimental results reveal that the LIC-1% H_3_PO_4_ sample, synthesized with 1% H_3_PO_4_, exhibits the lowest crystalline water content and the highest ionic conductivity of 1.15 × 10^−3^ S/cm. When assembled into ASSLIBs with the LiCoO_2_-LIC/LIC-1%H_3_PO_4_/LPSC/In/Li configuration, the battery achieves stable cycling for 14 cycles at 30 °C, delivering a first-cycle charge capacity of 128 mAh/g. Additionally, Li-In/LPSC/LIC-1% H_3_PO_4_/LPSC/Li-In symmetric cells demonstrate stable cycling over 1000 cycles under current densities of 0.05, 0.1, and 0.3 mA/cm^2^. Mechanistic studies indicate that PO_4_^3−^ in the solution thermally decomposes into P_2_O_7_^4−^ at elevated temperatures, ultimately forming LiIn(P_2_O_7_) with a vacancy-rich framework that facilitates Li^+^ transport, thereby enhancing the ionic conductivity of LIC. This work highlights that LIC synthesized with 1% H_3_PO_4_ achieves optimal performance and elaborates on how the P_2_O_7_^4−^ anion (derived from H_3_PO_4_) creates a vacancy-rich framework in LiIn(P_2_O_7_) (Figure 8a), facilitating Li^+^ transport and suppressing interfacial side reactions (e.g., In_2_O_3_ formation), offering a low-cost and scalable strategy for producing high-conductivity LIC electrolytes.

## Figures and Tables

**Table 1:** Ionic conductivity of LIC prepared in different concentrations of H_3_PO_4._

Sample	Conductivity σ (×10^−3^ S/cm)
LIC-H_2_O	0.38
LIC-0.5% H_3_PO_4_	0.65
LIC-1% H_3_PO_4_	1.15
LIC-2% H_3_PO_4_	0.53

## Data Availability

The original contributions presented in the study are included in the article, further inquiries can be directed to the corresponding authors.
